# Kita Driven Expression of Oncogenic HRAS Leads to Early Onset and Highly Penetrant Melanoma in Zebrafish

**DOI:** 10.1371/journal.pone.0015170

**Published:** 2010-12-10

**Authors:** Cristina Santoriello, Elisa Gennaro, Viviana Anelli, Martin Distel, Amanda Kelly, Reinhard W. Köster, Adam Hurlstone, Marina Mione

**Affiliations:** 1 IFOM Foundation – FIRC Institute of Molecular Oncology, Milan, Italy; 2 Helmholtz Zentrum München, Institute of Developmental Genetics, Neuherberg, Germany; 3 Faculty of Life Sciences, University of Manchester, Manchester, United Kingdom; Universidade de São Paulo, Brazil

## Abstract

**Background:**

Melanoma is the most aggressive and lethal form of skin cancer. Because of the increasing incidence and high lethality of melanoma, animal models for continuously observing melanoma formation and progression as well as for testing pharmacological agents are needed.

**Methodology and Principal Findings:**

Using the combinatorial Gal4 –UAS system, we have developed a zebrafish transgenic line that expresses oncogenic HRAS under the *kita* promoter. Already at 3 days transgenic *kita*-GFP-RAS larvae show a hyper-pigmentation phenotype as earliest evidence of abnormal melanocyte growth. By 2–4 weeks, masses of transformed melanocytes form in the tail stalk of the majority of *kita*-GFP-RAS transgenic fish. The adult tumors evident between 1–3 months of age faithfully reproduce the immunological, histological and molecular phenotypes of human melanoma, but on a condensed time-line. Furthermore, they show transplantability, dependence on *mitfa* expression and do not require additional mutations in tumor suppressors. In contrast to *kita* expressing melanocyte progenitors that efficiently develop melanoma, *mitfa* expressing progenitors in a second Gal4-driver line were 4 times less efficient in developing melanoma during the three months observation period.

**Conclusions and Significance:**

This indicates that zebrafish *kita* promoter is a powerful tool for driving oncogene expression in the right cells and at the right level to induce early onset melanoma in the presence of tumor suppressors. Thus our zebrafish model provides a link between *kita* expressing melanocyte progenitors and melanoma and offers the advantage of a larval phenotype suitable for large scale drug and genetic modifier screens.

## Introduction

Melanoma development has classically been described as a stepwise process in which mature melanocytes acquire increasing number of mutations in oncogenes or tumor suppressor genes leading to uncontrolled proliferation, acquisition of invasive properties and metastatic potential [1]. However the progression from benign nevi, radial growth, vertical growth and metastatic melanomas is not a universal feature of all melanomas, with more than 50% of the tumors originating from normal skin, rather than from dysplastic nevi [2]. This data suggest that although melanoma appears to be due to the transformation of mature melanocytes, it may derive from melanocytic progenitor or stem cells that upon transformation become able to initiate and maintain cancer development.

The identification of melanoma initiating cells (MICs) is of paramount importance to devising methods for early detection and eradication of the disease. To this end, the most valuable tools are in vivo models of melanoma that can be used to study the cell of origin of the disease, the mechanisms of transformation and the markers that distinguish the progressive changes in tumor cells and monitor/target them at early stages.

A number of genetic mouse models have been engineered to express oncogenes in melanocytes [3]. Regardless of the oncogene used, the presence of inactivating mutations of tumor suppressors or other genetic modifiers, these transgenic models use the promoter of the *tyrosinase* (*tyr*) gene, which is expressed in melanocytes, but not in their progenitors, thus practically selecting the population of cells to transform. Furthermore, the large size and inaccessibility of mouse embryos developing in utero make it difficult to address early developmental processes of melanocyte transformation.

Zebrafish embryos are small in size, develop outside their mother and are nearly transparent allowing for melanocytes to be followed during all stages of their development. This has been exploited for establishing melanoma models in zebrafish, which coupled with the ease of administration of chemicals through the water make them ideal for drug screening [4]. All the models developed so far use the promoter of the *mitfa* gene [5], [6], [7], [8] to drive oncogene expression. In these models, with the exception of the model developed in medaka using the *Xmrk* oncogene [7], melanoma develops only in the presence of coadjuvating mutations in p53 [5], [8] or if they do arise spontaneously then again take several months to materialize and then only infrequently [6].

Here we report on a zebrafish model of melanoma that expresses oncogenic HRAS under the *kita* promoter and develops melanoma by 1–3 months of age, without the need of coadjuvating mutations in tumor suppressors

## Results

### Expression of oncogenic HRAS driven by kita promoter/enhancer sequences induces hyperproliferation of embryonic melanocytes

In order to generate a flexible zebrafish model of melanoma we took advantage of the UAS-Gal4 binary system, which has been extensively used in Drosophila and lately developed for the zebrafish [9], [10], [11], [12], [13].

**Figure 1:**
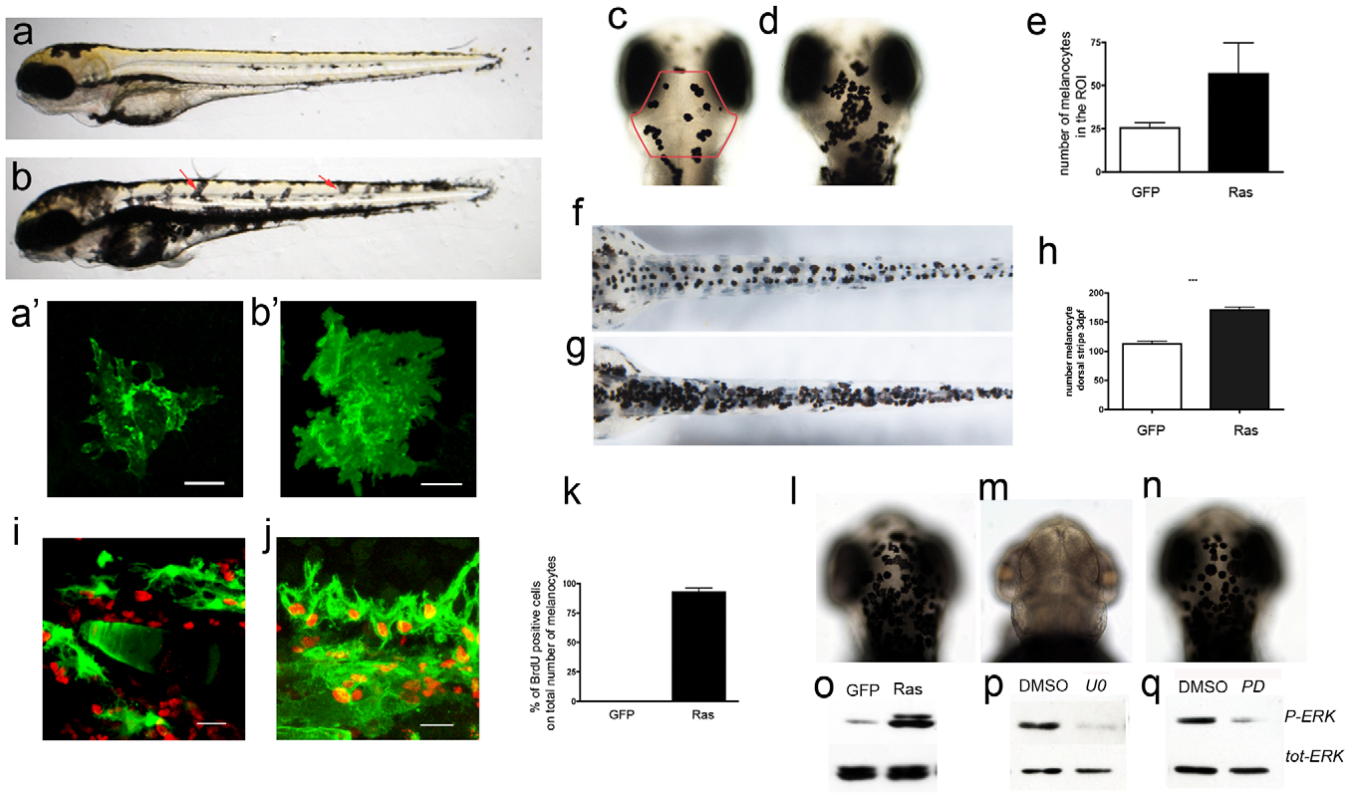
Expression of oncogenic HRAS driven by *kita* promoter induces hyperpigmentation in larvae. a) A 3dpf wild-type zebrafish; b) a kita-GFP-RAS hyperpigmented transgenic with displaced melanocytes (red arrows). A'-b') size and shape of representative head melanocytes in control (a') and kita-GFP-RAS embryos (b') c–e) Dorsal views of the head of control (c) and *kita*-GPF-RAS larvae (d). The red outline in (c) indicates the ROI where counts were made. e) Quantification of the number of melanocytes (mean±SD) (mean±SD, ***p<0.001) as indicated. f–h) Dorsal views of the lower body of control (f) and *kita*-GPF-RAS larvae (g). h) Quantification of the number of melanocytes (mean±SD, ***p<0.001) as indicated. i–k) GFP marked melanocytes (green) and BrdU immunostaining (red) in kita-GFP (i) and kita*-*GFP-RAS (j) 4dpf zebrafish. Calibration bar: 20 µm. k) Percentage of BrdU+ melanocytes in control (GFP) and melanoma line (Ras) at 4dpf (mean±SD, n = 5). l–n) Dorsal views of heads of *kita*-GFP-Ras larvae raised in DMSO (l), in U0126 (m) or in PD98059 (n) in the presence of epinephrine. o) WB analysis performed 3dpf GFP (control larvae) and RAS (kita-GFP-RAS larvae) revealed an activation of P-ERK. p–q) Treatment with UO126 (UO) or PD98059 (PD) caused a reduction of P-ERK. Calibration bars: 50 µm for a, b, c–f, l–n; 20 µm for i–j.

We crossed fish from *Et(kita:GalTA4,UAS:mCherry)hzm1* line [13] with fish from the *Tg(UAS:eGFP-H-RAS_GV12)io6* line [14] and observed that larvae expressing oncogenic HRAS in a pattern driven by *kita* regulatory sequences show an altered pigment pattern and increased pigmentation already starting from 3dpf (figure 1a, b). To simplify nomenclature, we will refer to larvae/adult from this cross as kita-GFP-RAS zebrafish and to controls, which derive from a cross between the *Et(kita:GalTA4,UAS:mCherry)hzm1* and the *Tg(UAS:eGFP)* (a gift of Masa Tada, UCL, see material and methods) lines, as kita-GFP zebrafish.

When kita-GFP-RASV12 embryos were analyzed for transgene activation, GFP fluorescence was observed in melanocytes throughout the embryo indicating the faithful cell type specific expression of the oncogene but also the possibility to identify transgenic cells in vivo. Individual melanocytes in the hyperpigmented larvae failed to localize along the horizontal stripes where they normally reside (figure 1b) and were significantly larger than in controls at 3 dpf (figure 1a', b').

In order to evaluate if the number was increased we counted melanocytes present in a region of interest (ROI) encompassing the dorsal pigment stripe between the eyes and the base of the head (outlined in figure 1c) and in the dorsal body stripe (figure 1f, g) in the presence of constant light for 3 days as this causes melanocyte contraction and facilitates counting [15]. Statistical analysis revealed that kita-GFP-RASV12 larvae have double the number of melanocytes (56.8±17.9 versus 25.6±2.8, n = 10) compared to control larvae (figure 1e, h). This suggests that the expression of the HRAS oncogene promotes growth and maintains melanocytes in a proliferative state, whereas normally many melanocytes are postmitotic by 3 dpf. We used BrdU to label melanocytes that are still proliferating at 3-4 dpf. Almost all melanocytes were BrdU labeled in kita-GFP-RAS larvae following 12 hrs exposure to BrdU at 3-4 dpf (figure 1j, k), whereas very few (less that 5% of all melanocytes) were BrdU labelled in kita-GFP larvae (figure 1i).

To test if the hyperpigmentation phenotype was due to an activation of the MAP kinase pathway downstream of ras signaling, we checked for ERK1/2 phosphorylation using Western Blot analysis. An increase in the levels of phospho ERK was found in kita-GFP-RAS larvae compared to controls (figure 1o). Accordingly, treatment of kita-GFP-RAS larvae with UO126 (an inhibitor of MEK1/2 and a copper chelator, [16] caused a corresponding decrease of ERK1/2 phosphorylation (figure 1p) and reduced hyperpigmentation to different degrees (figure 1m). In contrast to UO126, treatment with PD98059 (which efficiently inhibited ERK1/2 phosphorylation in kita-GFP-RAS larvae, figure 1q) was unable to prevent hyperpigmentation (figure 1n). This suggests that hyperpigmentation caused by melanocyte hyperplasia in kita-GFP-RAS larvae is not due to ERK1/2 phosphorylation and is dependent on copper uptake (as in control melanocytes [16]).

### Kita-GFP-RAS fish develop melanoma at early stages, and these tumors are similar to human melanoma

**Figure 2:**
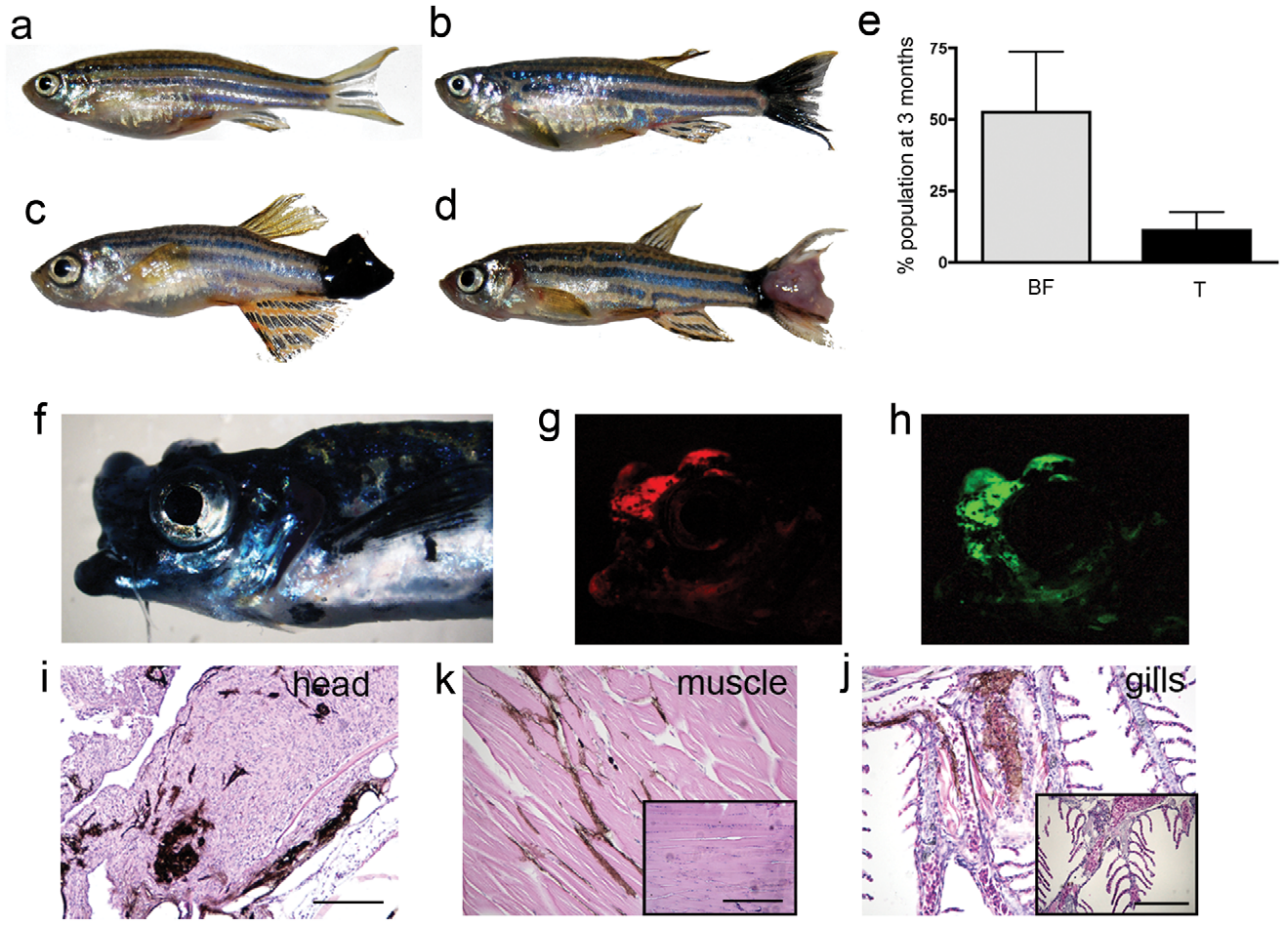
kita-GFP-RAS fish develop melanoma. a–d) Different phenotypes in three-month old kita-GFP-RAS zebrafish: a) control fish; b) black caudal fin; c) large hyper-pigmented tumor; d) hypo-pigmented tumor. e) Percentage of the progeny of kita-GFP-RAS fish incross displaying a black fin phenotype (BF) or tumor (T) at 3 months (mean±SD). f) Lateral view of a fish bearing an infiltrating melanoma of the mouth and head, which also expresses mCherry (g) and GFP-RASV12 (h). *H&E* staining of parraffin sections of the fish in f) that display infiltrations of melanocytes in the head (i), muscles (k), gills (j). Insets are from control animals. Calibration bars for i–j  = 100 µm.

Zebrafish undergo a change in pigmentation pattern around 2–3 weeks of age. This period is known as metamorphosis and leads to the mature pigment pattern of the adult fish, which is established around 4 weeks of age [17], [18]. Kita-GFP-RAS fish develop an accumulation of melanocytes at the level of the caudal fin that often cause disruption of normal tissue surrounding these lesions that we call melanocytic hyperplasia (figure s1) starting from 2 weeks post fertilization. These lesions occur in almost 100% of double transgenics before metamorphosis (melanocytic hyperplasia and in some cases juvenile tumors, see figure s1e), but then not all of these lesions progress and we could only find a black caudal fin after metamorphosis in several kita-GFP-RAS transgenic fish at 1 month (figure s1) or 3 months (figure 2b, e). Notably though in approximately 20% of 1–3 month old kita-GFP-RAS fish (n = 252), tumors progress to an invasive phenotype (figure 2c, figure s1e, o). Most of these adult tumors are hyper-pigmented, with a very low percentage of them (1–2%) being hypo-pigmented (figure 2d).

In order to evaluate whether these tumors were invasive we performed histological analysis on a collection of fish displaying tumors. In figure 2f we show a fish displaying a tumor at the level of the mouth, which is also characterized by the expression of Gal4-mCherry (figure 2g) and GFP-HRASV12 (figure 2h). We observed that melanocytes were infiltrating various tissues/organs including brain (figure 2i), muscles (figure 2k) and gills (figure 2j).

Histology performed on 2 week-, 4 week- and 2 month-old kita-GFP-RAS fish showed progressive infiltration of tail tissue by transformed melanocytes, (figure S2a, b) up to a complete substitution of tail tissue with melanoma tissue (figure s2c).

Both hyper- and hypo-pigmented tumors are enriched in melanocytes as revealed by EM analysis of tumor samples (figure s2d, e) and in situ hybridization on cryostat section for *dopachrome tautomerase (dct*, a marker of melanin biosynthetic pathway [19], figure s2f).

To assess how useful this model could be to study the biology of human melanoma progression we investigated the immunological features of the zebrafish tumors using the same markers that are used in diagnostic for human melanoma.

**Figure 3:**
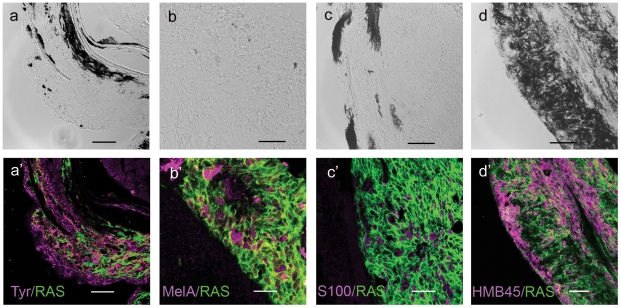
Immunostaining of zebrafish melanoma cryostat sections. In the lower panels (a'–d') immunostainings (magenta) for the markers indicated in the lower right corners of each micrograph. All cells express GFP-RAS (green at the plasma membrane). Upper panels (a–d) show the DIC image of each sample, which consisted mostly of unpigmented tumors. Calibration bars  = 50 µm.

Immunostaining on zebrafish melanoma cryostat sections including Tyrosinase [23], Melan-a [22], s100 [21] and HMB45 [6], [20], revealed elevated expression of all these melanoma markers showing that these tumors are immunologically similar to human melanoma (figure 3).

**Figure 4:**
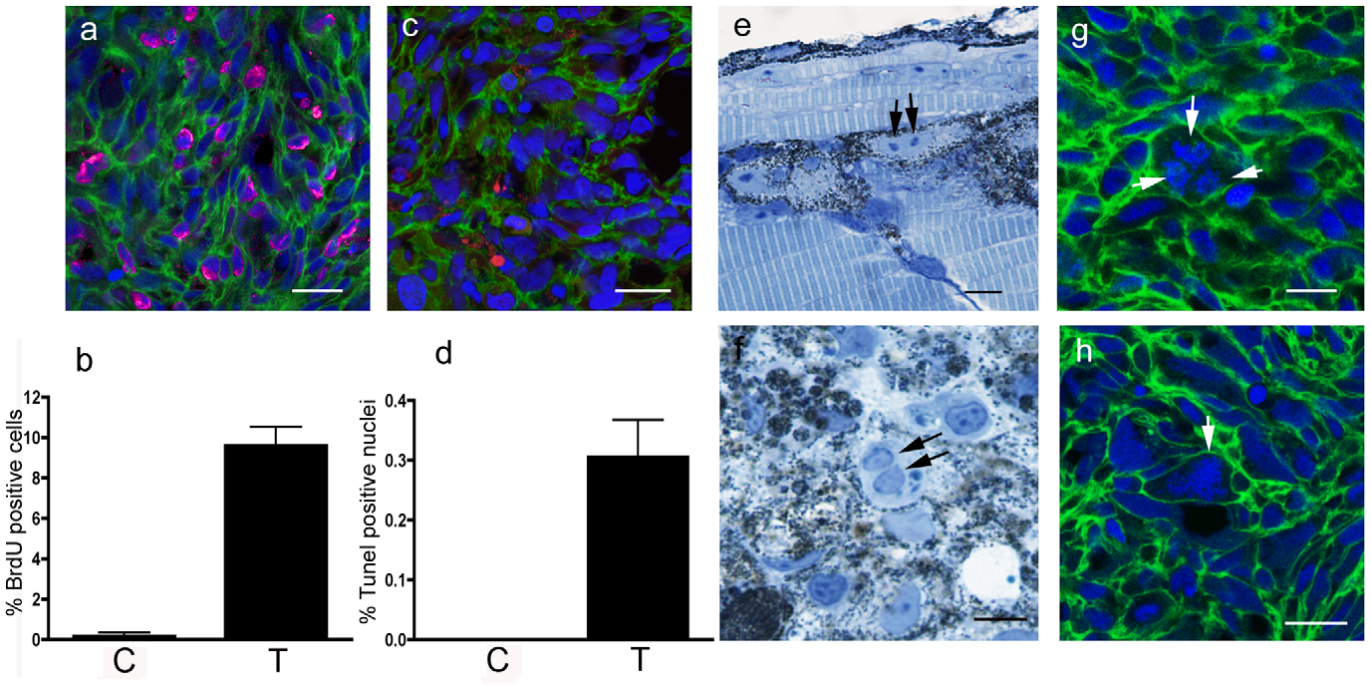
Histology of kita*-*GFP-Ras melanoma. a) BrdU+ nuclei (magenta) in a section of a melanoma developing in a 3-month-old kita-GFP-RAS fish. b) Diagram showing percentage of BrdU+ cells in control skin (C) and melanoma (T) sections (n = 5). c) TUNEL+ nuclei (red) in a cryostat section of a melanoma developing in a 3-month-old kita-GFP-RAS fish. d) Diagram showing percentage of TUNEL+ cells in control skin (C) and melanoma (T) sections (n = 5). e) Semithin (0.5 µm thick) resin section of a 5dpf kita-GFP-RAS larva, showing several melanocytes, one of which is at the end of mitosis. Arrows point to the two chromosomal condensations. f) Semithin section of a tumor from a 3-month-old kita-GFP-RAS fish showing enrichment in melanocytes one of which is binucleate (arrows). g–h) Cryostat sections of melanomas developing in kita-GFP-RAS 3-month-old fish. All cells express GFP-RAS (green at the plasma membrane) and have DAPI (blue) counterstained nuclei. Arrows point to a multipolar spindle (g) and a polyploid giant nucleus with condensed chromosomes (h). Calibration bars: 50 µm.

We also performed an analysis of the proliferation, apoptosis and presence of nuclear abnormalities in 5 different melanoma samples and found that approximately 10% of melanoma cells are dividing (figure 4a, b), whereas less than 1% of tumor cells undergo nuclear fragmentation and show TUNEL labeling (figure 4c, d); however, nuclear abnormalities were common in tumor cells and dividing melanoblasts or binucleate melanocytes were found in semithin sections of hyper-pigmented transgenic larvae (figure 4e) and melanoma (figure 4f). Furthermore, we detected nuclear abnormalities including multipolar spindles (figure 4g) and polyploid nuclei (figure 4h) as commonly found in melanoma cell lines.

### Tumor formation does not require mutations in p53 or PTEN

**Figure 5:**
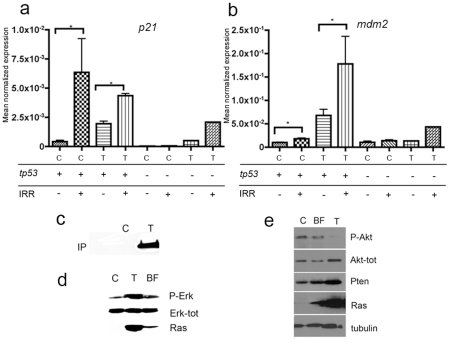
kita-GFP-Ras tumors have a robust p53 pathway activation. a–b) QPCR analysis of tp53 target gene expression. *p21*(a) and *mdm2* (b) mRNA expression in control (C) or tumors from *kita-*GFP-RAS transgenic fish (T) with a wild-type (*tp53+*) or mutant (*tp53 -)* background genotype, as indicated. Samples were subjected to X-ray irradiation (IRR +) or left unirradiated (IRR -). All data are expressed as Mean Normalized Expression and were normalized to the expression levels of β-actin as an endogenous control. Error bars indicate s.d. except for *tp53*-/-, for which the error bars indicate range. n = 3 for C *tp53*+, IRR-; n = 3 for C *tp53*+, IRR+; n = 9 for T *tp53*+, IRR-; n = 3 for *tp53*+, IRR+; n = 2 for C *tp53*- IRR-; n = 2 for C *tp53*- IRR+; n = 1 for T *tp53*- IRR-; n = 1 for T *tp53*- IRR+. c-e) RAS pathway activation in tumor. c) Protein extracts from control and fin with tumor show active ras (Ras-GTP, immunoprecipitated with Raf1). Western Blot analysis of protein extracts prepared from control (C), fin with tumor (T) and black fin (BF) show an increased phosphorylation of ERK1/2 (d) and increased amounts of total AKT, but not phospho-AKT, which correlates with an increase in PTEN levels (e).

In two of the zebrafish melanoma models published so far [8] [5] it has been shown that tumor formations occurs only in the presence of inactivating mutations in *tp53*. To check whether tumor formation in our model is also due to an intervening mutation in *tp53*, we evaluated the ability of p53 to signal transcriptionally following X-ray treatment of kita-GFP-RAS or control fish by looking at the expression of two p53 downstream targets, p21 and mdm2 [24]. Irradiation causes upregulation of p53 targets and thereby we evaluated the levels of p21 and mdm2 expression in tumors deriving from kita-GFP-RAS irradiated fish. The levels of p21 and mdm2 expression were found significantly increased in quantitative PCR (figure 5a, b) indicating that p53 is trascriptionally active in these tumors. We also grew kita-GFP-RASV12 transgenic fish in a *tp53-/-* background [25]. We observed that these fish had a much faster progression of melanoma, requiring euthanasia at 1–3 months (not shown). Irradiation of *tp53-/-* control fish did not cause any increase in the levels of p21 and mdm2 expression. In tumors from kita-GFP-RAS; *tp53-/-* zebrafish the levels of p21 and mdm2 expression, are slightly increased after irradiation, suggesting that the residual activation of p21 and mdm2 may be p53-independent (as reported for other malignancies [26]). This suggests that tumor formation in our model due to oncogenic HRAS takes place in the presence of transcriptionally active p53. However, these results also suggest that melanoma development is further enhanced by the lack of p53 activity indicating a role for p53 as modulator of melanoma formation in our genetic model.

It has been reported that in melanoma both the Ras/Raf/MEK/ERK (MAPK) and the PI3K/AKT (AKT) signalling pathways are constitutively activated through multiple mechanisms [27], [28]. In order to evaluate whether these pathways are hyperactivated in our melanoma model we performed Western blot analysis on protein extracts prepared from control fin, black fin and fin with tumors. We found a robust activation of the ERK pathway (figure 5c, d) but we did not observe any increase of phospho AKT (figure 5e) in spite of increased levels of total AKT in tumors. Since there is an inverse correlation between PTEN and AKT activation [29] we thought that AKT might be indirectly dephosphorylated by PTEN with a very quick kinetic. In order to test this hypothesis we checked the expression levels of PTEN by Western blot analysis, and found elevated levels of PTEN in tumor samples (figure 5e).

These results support the hypothesis that in our model, tumors may develop in the presence of PTEN or p53 tumor suppressor activities.

### Melanoma development is dependent on mitfa expression

**Figure 6:**
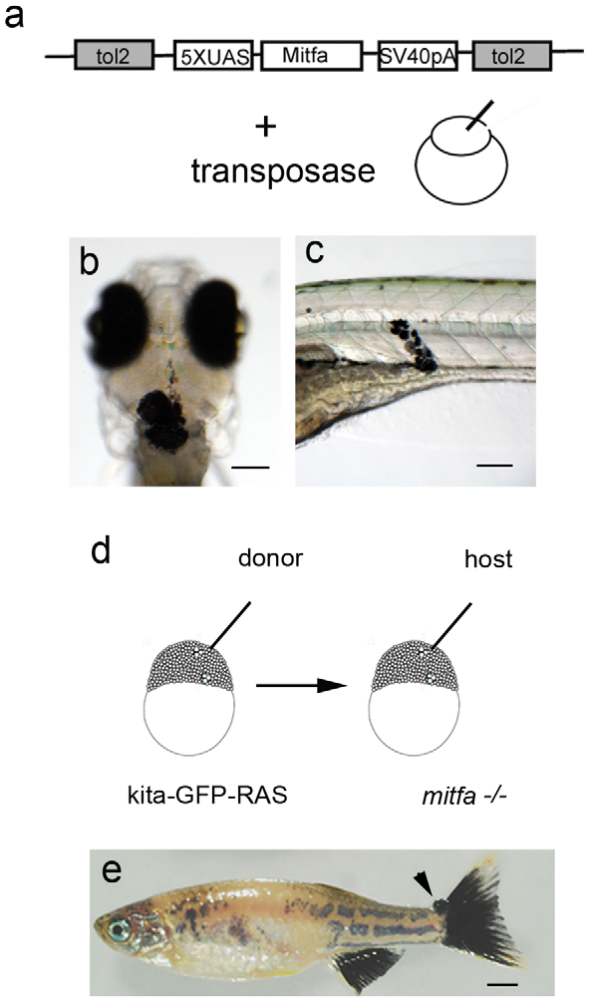
Melanoma development is dependent on mitfa expression. a) Schematic diagram of the construct and injection strategy used to re-express mitfa in *mitfa-/- (nacre);* kita-GFP-RAS zebrafish. Transformed melanocytes in the head of a 30hpf (b) and in the trunk of 5dpf (c) *mitfa-/- (nacre);* kita-GFP-RAS injected larvae. d) Schematic representation of the transplantion strategy, where cells were transplanted from kita-GFP-RAS embryos into *mitfa-/- (nacre)* embryos at approximately 1000 cell stage. e) Picture of 4 month-old trasplanted *mitfa-/- (nacre)* fish displaying a patchy recovered pigment pattern and a small tumor at the level of the caudal fin (arrow). Calibration bars = 100 µm for b,c; 2 mm for e.

We investigated whether the formation of melanoma in kita-GFP-RAS transgenic fish relies on the expression of *mitfa,* the master regulator of melanocyte survival and differentation [30]. We raised the kita-GFP-RAS line in a *mitfa-/-* background (a mutant called nacre [31]) and observed that these embryos, although initially expressing the GFP:HRASV12 transgene in melanocyte progenitors (figure s3a) by 3 dpf were devoid of melanocytes (figure s3b, e) and did not develop any tumor by 9 months of age. To further confirm that the expression of *mitfa* is required for melanocyte survival and melanoma development in double transgenic fish we mosaically re-expressed *mitfa* in kita-GFP-RasV12/nacre embryos. To do so, we cloned the *mitfa* coding region in a UAS expression vector (figure 6a) and injected this construct in kita-GFP-RASV12/*nacre* embryos at one cell stage. We observed already at 30hpf the presence of melanocytes (figure 6b), able to proliferate and form clones (figure 6c), which survived at least for a few weeks. This suggests that, as reported by a large body of literature [30], [32] *mitfa* expression is necessary for melanocyte survival and the expression of HRASV12 is not sufficient to rescue them.

We then used transplantation in *nacre* fish to investigate if the oncogene activity is cell-autonomous and whether its expression confers melanocytes the ability to survive and positively compete with host cells. To do so we transplanted cells from kita-GFP-RAS (donor) into *nacre* or wild type (AB) embryos (host) at blastula (figure 6d, e). We raised all the transplanted fish and we observed that 57% of *nacre* fish receiving cells from kita-GFP-RAS donors developed tumors associated with a black caudal fin phenotype (figure 6e). In AB hosts, donor kita-GFP-RAS cells survived and proliferate to generate melanocytic hyperplasia in a similar percentage of cases (49%). This result suggests that kita-GFP-RAS cells maintain their transformed and aggressive phenotype in a completely cell-autonomous fashion, and that ras-expressing melanocytes survive and thrive equally well in the presence or in the absence of competition from host melanocytes. Thus, a large percentage of kita-GFP-RAS expressing cells is able to initiate melanoma development in a host environment.

### Expression of HRASV12 under the mitfa promoter does not reproduce the kita-GFP-RAS phenotype

Altogether, these results suggest that targeting the expression of the HRAS oncogene to a population of cells that express the kita gene is able to induce melanoma development with high efficiency (i.e. without the need for additional mutations that inactivate tumor suppressors) and in a relatively short time (2–4 weeks). We hypothesize that the aggressive features of our model depends not so much on the oncogene which has been used also in another zebrafish model of melanoma [6], but rather on the cell types that are targeted by the kita promoter perhaps also in conjunction with higher levels of oncogene expression.

**Figure 7:**
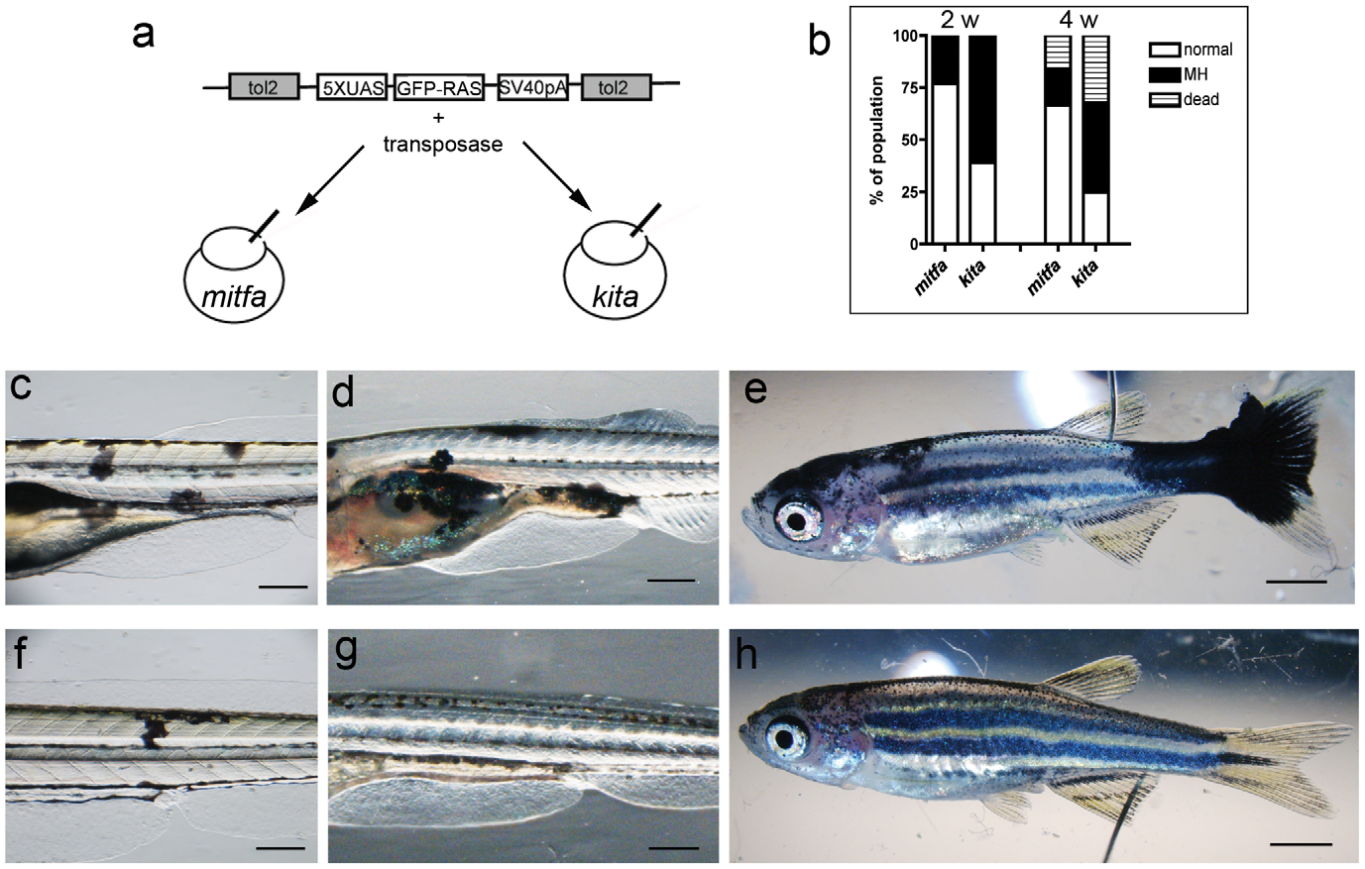
Comparison between *mitfa* and *kita* Gal4 driver lines. a) Schematic diagram of the construct and injection strategy used to transiently express GFP-HRASV12 in the *mitfa:Gal4* and *kita:Gal4* lines. b) Percentage of injected fish that display melanoytic hyperplasia/melanoma (MH) at 2 and 4 weeks (w) post fertilization. c–e) 4, 14 and 32dpf *kita-Gal4* fish injected with *UAS-GFP-HRASV12* plasmid for somatic integration. Mosaic expression of the transgene leads to abnormal melanocytes (c), persistent melanocytic hyperplasia (d) and melanoma (e) f–h) 4, 14 and 32dpf *mitfa-Gal4* fish injected with *UAS-GFP-HRASV12* plasmid. Mosaic expression of the transgene in this driver line generates fewer and smaller melanocytic lesions. Calibration bars  = 2 mm.

We tested the ability of the *UAS:HRASV12* transgene to induce melanoma development following expression in somatic cells (i.e. in F0, after injection of the *UAS:GFP-HRASV12* plasmid in the *tg(mitfa:Gal4VP16; UAS:mCherry)* line - named mitfa:Gal4 to simplify, or in the kita:Gal4 line, figure 7a). This approach is commonly thought to yield high level of expression. Here we evaluated abnormal melanocyte proliferation at 4dpf (figure 7c, f), 15dpf (figure 7d, g) and transformation at 1 month (figure 7e, h) in larvae/juveniles that were selected for 1 or more transient integration events (i.e. displaying GFP+ cells) at 2 dpf. At 4 dpf lesions in both lines (n = 132 in mitfa:Gal4 and n = 110 in kita:Gal4) were composed of several melanocytes, indicating that the oncogene stimulates proliferation and supported the clonal expansion of the cell carrying somatic insertion of the oncogene (figure 7c, f). At 15 dpf 57.3% of the melanocytic lesions in the *Et(kita:GalTA4,UAS:mCherry)hzm1* (39 out of 68 survivors) were still expanding (figure 7b, d), whereas only 17.2% were detectable in the mitfa:Gal4-mCherry line (16 out of 93 survivors, figure 7b, g). At 1 month 50% of the kita:Gal4-mcherry HRASV12 injected fish showed clear malignant melanoma (22 out of 44 survivors, figure 7b, e), whereas melanomas were present in only 11% of the mitfa:Gal4-mcherry HRASV12 injected fish (9 out of 83 survivors, figure 7b, h), indicating that many melanocytic lesions present at 15 dpf had regressed. We also compared melanoma development in double transgenic lines obtained from mitfa:Gal4 or kita:Gal4 crossed to the same UAS:GFP-HRASV12 reporter line. The ras oncogene was expressed in a similar pattern in migrating neural crest cells in both mitfa-GFP-RAS and kita-GFP-RAS double transgenic embryos at 30 hpf (but in fewer cells in the mitfa-GFP-RAS line figure s4a, b), but the hyper-pigmentation phenotype does not develop in the mitfa:GFP-RAS larvae (figure s4c). We observed tail melanocytic hyperplasia in 3 out of 25 double mitfa-GFP-RAS transgenics at 24 dpf (which regressed completely by 34 dpf) and one case of a head melanoma at 3 month of age (in 25 double transgenic adult fish, figure s4d).

To understand the reasons of the difference between mitfa and kita driver lines in developing melanoma, we studied the cell types that express the oncogene under the control of the two driver lines. We found that in kita-GFP-RAS embryos and larvae other cell types not previously associated with the melanocytic lineage express the oncogene. None of these cell types display features of melanoblasts (being mostly terminally differentiated cells, see figure s4g, h). However, there is a possibility that these cell types share the same lineage of melanocytes and that the kita-GFP line may provide insights on this. We then investigated if the differences between the two driver lines are due to different level of HRAS being expressed or maintained in melanocytes using western blot analysis, and found that in the mitfa-GFP-RAS line the levels of RAS expression are very low compared with those found in kita-GFP-RAS larvae and adults (fig. s4i). This result suggest that the higher penetrance and earlier onset of melanoma in the kita-GFP-RAS line versus mitfa-GFP-RAS line could be due to the levels and persistence of oncogene expression, rather than to different cell specificity of the two promoters.

## Discussion

Here we report on a genetic, inheritable zebrafish model of melanoma, which has a number of properties providing insights and tools for the study of melanoma biology and that shows features comparable to human melanoma.

First, this model shows that expression of oncogenic HRAS can initiate and maintain melanoma formation without the need for inactivating mutations in tumor suppressors as reported for other (zebrafish and mouse) models of melanoma [8], [33]. Second, the presence of a larval phenotype that is a direct precursor of the melanoma lesions that develop at later stages, allows rapid, easy to score and specific chemical screens aimed at finding compounds and drugs that may revert the hyper-pigmentation phenotype. Third, the model provides a rapid approach to gene manipulation specifically in the HRAS transformed cells, that could be exploited for large scale suppressor (and enhancer) screens, through the use of UAS elements to drive expression of cDNA libraries, or for validation of drug target candidates. Fourth, the model allows to image cancer cells in vivo thanks to the expression of GFP.

Genetic melanoma models which reproduce the human phenotype and provides a source of stageable cancer samples are strongly needed to test hypotheses on the mechanisms of malignant transformation, identify melanoma initiating cells and study their features in order to devise way of eradicating them. The presence of well-defined intermediate stages of cancer development will provide the blueprint for marker/signature analysis of transcriptome, epigenome and proteome [34]. Even more ideal would be a flexible model suitable for genetic manipulation, *in vivo* imaging and pharmacogenomic approaches. Our model is amenable to all these approaches and in the following paragraphs we will briefly discuss the main findings obtained with this versatile melanoma model.

### Larval hyperpigmentation is an early sign of melanoma development and can be used in pharmacological screens aimed at finding anti-melanoma drugs

One of the features of our model is the development of a hyper-pigmentation phenotype at early larval stages which is suitable for large scale chemical screens aimed at discovering drugs that controls melanocyte number, migration and transformation. One important question here is whether larval hyperpigmentation in our model is associated with melanoma development. Hyperpigmentation has been reported in larvae overexpressing kit ligand (kitL,[35]. The hyperpigmentation phenotype due to overexpression of kitL resemble a human condition known as Familial Progressive Hyperpigmentation (FPH, MIM #145250) which is also due to gain of function mutations in KIT ligand [36] and has not been associated with melanoma development. From these data it appears that c-kit signaling controls proliferation, size and migration of melanoblasts and melanocytes. However an increase of kit signaling is not *per se* able to induce transformation of these cells and to sustain melanoma development [36]–[37]. Conversely, congenital nevi in children frequently harbor RAS, but not BRAF mutations [38] and are associated with an increasing risk of melanoma development. In our model the expression of oncogenic ras in larval melanocytes or their precursors is able to induce them to proliferate, subvert their interactions with neighbouring cells promoting multilayer growth, causes changes in shape and migration, induces polyploidy and alter their normal development. Both somatic and germline expression of HRASV12 in *kita+* cells can induce melanoma development from larval stages. This observation suggests that there is a continuum between transformed larval melanocytes and various grades of melanocytic lesions up to melanoma, which makes the larval hyperpigmentation phenotype a truly pre-melanoma phenotype. The parallels between congenital RAS-induced nevi and the clusters of transformed melanocytes in transgenic larvae suggest that the kita-GFP-RAS line could be a useful model for this condition.

### Unique advantages of the kita-GFP-RAS melanoma model

The zebrafish melanoma model described here has unique advantages over other zebrafish and mouse models of melanoma. First, the expression of a GFP tagged oncogene in melanoblasts offers the possibility of visualizing ras-transformed cells *in vivo* from the earliest stages of transformation. This is a great potential of the model making it accessible for non-invasively tracing of transformed melanocytes. Other aspects that can be studied *in vivo* using this model are the ability of ras transformed cells to escape immune-surveillance [39] or to induce favorable changes in the microenvironment that could promote the survival and expansion of clones of melanoma cells.

A second advantage of the melanoma line described here is the possibility of manipulating gene expression specifically in the transformed cells, thanks to the use of UAS elements to drive the expression of genes in the same cells that express oncogenic ras.

### The problem of melanoma initiating cells and the role of inactivating mutations in tumor suppressors

One of the most debated concepts in cancer research is the ability of oncogenes to transform. Decades of studies have revealed that not all cells respond to oncogenes by generating cancer. Very efficient tumor suppressor mechanisms exist that control the cell response to oncogenes, making them harmless, and these include apoptosis and cellular senescence (reviewed in [40]).

This is why most tumors develop only following two causative hits: firstly a mutation that transform a cellular gene in an oncogene, and secondly, a mutation that inactivate a tumor suppressor [41]. The need for the occurrence of two mutations may be bypassed by different mechanisms: on one side levels and activity of the oncogene may be so high, and the oncogene may be expressed in many cells (as in genetic cancer predisposition syndromes due to germline mutations) that neither apoptosis nor cellular senescence can get rid of all the transformed cells. Another mechanisms could be intrinsic to the cell type where the activating mutation takes place.

A growing body of evidence (reviewed in [42]) suggests that tumor suppressors are less active in stem/progenitor cells, as their activity would eliminate a multipotent progenitor, with very disruptive consequences for the whole organism. Mutations that inactivate tumor suppressors may be seen as absolutely necessary in the oncogenic transformation of terminally differentiated cells, while they may not be necessary to induce transformation in a multipotent progenitor, where tumor suppressors are less active.

In this study we have shown that *kita* expressing melanoblasts can be efficiently transformed by the HRAS oncogene in the presence of active p53 and give rise to melanoma with a higher efficiency and much lower latency than *mitfa* expressing melanoblasts and melanocytes. This difference may be due to the higher levels of HRASV12 expression driven by the *kita* and/or to different cell specificity of the *kita* and *mitfa* promoters. We have also observed the development of tail melanocytic hyperplasia in the majority of ras expressing metamorphic larvae suggesting that *kita-*expressing melanoblast progenitors may concentrate/reside in this location making it a preferential site for melanoma development. While this location seems to be specific for the fish, the occurrence of certain types of melanoma in humans in preferential locations (acral and mucosal melanoma, reviewed in [43]) may suggest a similar developmental mechanism (i.e. residence of melanoblast stem cells) that would be worth investigating.

In conclusion, we have reported here on a new model of melanoma in zebrafish, which provides new tools and new insights for the study of the biology of melanoma cells. It also holds the promise that the similarities between fish and human melanoma go beyond anatomical and developmental specificities, thus facilitating the use of this genetic model for human disease study and treatment.

## Methods

### Zebrafish strains and maintenance

Adult zebrafish (Danio rerio) were maintained as described [44]. Strains included: AB; *tg(UAS:GFP)* (a kind gift of Masa Tada, UCL), *tg(5XUAS:eGFP-HRASV12)io6* [14], *mitfa^w2/w2^* [31], *p53^zdf1/zdf1^* [25] and *tg(mitfa:Gal4VP16;UAS:mCherry)*; Et(*kita:GalTA4,UAS:mCherry*)*hzm1* [*13*]. The double transgenic line *Et(kita:GalTA4,UAS:mCherry)hzm1xtg(UAS:eGFP-H-RASV12)io6* was also raised in *tp53*-/- background [25]. Work with zebrafish was carried out under EU regulations for animal experimentation. The project was approved by Ministero della Salute - Direzione Generale della Sanità Animale e Del Farmaco Veterinario-Ufficio VI°, protocol number 02/2010 (under decree 116/92).

### Generation of driver and reporter lines

The *tg(UAS:eGFP-HRAS_G12V)io6* line was generated as described (19). For the generation of *tg(mitfa:Gal4VP16, UAS:mCherry)* line the *mitfa:Gal4VP16, UAS:mCherry* transgene was constructed by inserting a *mitfa* proximal promoter fragment [6] before a *Gal4VP16-15XUAS* tandem self-reporting fragment (a kind gift of Michael Parsons), itself placed before intron 1 of the zebrafish *EF1alpha* gene combined with *mCherry* cDNA. The plasmid backbone was *pT2AL200R150G* (a kind gift from Koichi Kawakami). The construct was injected into 1-cell stage zebrafish embryos and a transgenic line with neural crest restricted expression expanded.

### Live imaging of transgenic zebrafish

Anaesthetised transgenic larvae were embedded in 1% agar and viewed with a Leica SP2 confocal microscope.

### Melanocyte quantitation

Embryos were raised in different light regimes from birth till 3dpf. They were fixed in 4% paraformaldehyde overnight, and then transferred into phosphate-buffered saline containing 10% Tween-20. Pigment cells in the dorsal pigment stripe on the anterior of the embryos were imaged using a up-right microscope (Nikon,Tokio Japan). We manually counted the number of melanocytes in the region of interest using the NIS-elements software (Nikon).

For epinephrine treatment (to cause contraction of melanocytes) embryos were exposed to epinephrine (Sigma) 1 mg/ml for 10 minutes and then fixed in cold 4% paraformaldehyde.

### BrdU labeling and TUNEL assay

For BrdU labeling, we incubated 72hpf larvae or adult fish in 10 mM BrdU in E3 fish water for 12 hours. Following fixation in 4% paraformaldehyde, larvae were treated with 2N HCL for 20 minutes, before processing them for immunofluorescence with anti-BrdU antibody (Sigma). A Cy5 conjugated secondary antibody was used to reveal BrdU uptake. For the adult fish, tumors or corresponding healty skin from control fish was excised after a light fixation and maintained in 4% paraformaldehyde overnight, before equilibration in 25% sucrose in phosphate buffered saline (PBS) and embedding in OCT. Cryostat sections (12 mm thick) were treated with 2N HCL as above and processed for immunohistochemistry as described for whole mount larvae.

Apoptotic cells in larvae or adult fish were specifically detected by ApopTag Red In Situ Apoptosis detection kit (S7165, Chemicon), which is based on terminal transferase dUTP nick-end labelling (TUNEL) assay. Samples were mounted in Vectashield (Vector Laboratories, Inc.) with DAPI.

Percentages of BrdU and TUNEL labelled melanocytes were evaluated in 6 controls (kita-GFP) and 6 kita-GFP-RAS larvae at 4 dpf after overnight exposure to BrdU. At least 200 melanocytes per speciments were evaluated for BrdU labelling. For tumor sections, counts refer to an area of 2.4 mm^2^. At least 3 sections, from 6 different tumors were analyzed.

### Immunofluorescence and antisera

Adult fish were killed by anesthetic overdose and tumors or corresponding areas from wild-type fish were excised and embedded unfixed in optimum cutting temperature (OCT; Sakura Finetek, Torrance, CA). Cryostat sections (14 µm thick) were fixed in 2% paraformaldehyde for 2 min. We used the following antisera: anti HMB 45 (1∶50 Novo Castra laboratories), and s100 (1∶50 Novo Castra laboratories), Melan-A (Novo Castra laboratories), and Tyrosinase (H-109) (1∶50 SantaCruz). Secondary antibodies conjugated with Alexa633 were from Molecular Probes (Carlsbad, CA). 4′,6-Diamidino-2-phenylindole (DAPI) counterstain was carried out on all sections. The expression of HRASGV12-GFP was still visible as green fluorescence.

### Western Blot analysis and Ras activation assay

Adult fish were killed by anesthetic overdose (0,04% MESAB, Sigma), the tumors were homogenized in sample buffer (2% SDS, 10% glycerol, 60 mM Tris pH 6.8). 20–50 µg of total extracts were resolved by SDS-PAGE, transferred to nitrocellulose and tested with the following antibodies: RAS (1∶500, BD bioscience), Phospho-p44/42 (1∶1000, Cell Signaling), p44/42 (1∶1000, Cell Signaling), Phospho-AKT (1∶1000, Cell Signaling), AKT (1∶1000, Cell Signaling), PTEN (1∶1000, Cell Signaling), Actin (MP Biomedical). We performed the StressXpress Ras Activation kit (Stressgen, Bioreagents) on protein extracts from larvae and juvenile fish according to the manufacturer's instructions.

### Drug treatment

Embryos starting from shield stage were incubated in Petri dishes with diluted compounds: 1 µg/ml of PD98059 (Calbiochem) or 10 µM of U0126 (Calbiochem) with 0.2% DMSO in E3 medium [45]. Embryos were kept in the dark to protect drugs from degradation.

### Histology and electron microscopy

Paraffin Embedding and H&E staining: the fish were dehydrated in alcohol, decalcificated in 0.25M EDTA, cleared in xilene and infiltrated with paraffin. Briefly slides were deparaffinized in xilene, passed throught graded alcohols, rinsed in water and briefly soaked in Hematoxylin and then in Eosin. Slides were dehydrated and mounted using EUKITT (GmbH & Co).

Five dpf larvae or tumor pieces were fixed in a mixture of 2% paraformaldehyde and 2% glutaraldehyde in 0.1 M cacodylate buffer (pH 7.4) over night, and postfixed in 1% osmium tetroxide for 1 hour. The specimens were then rinsed in buffer, dehydrated in a graded ethanol series and embedded in Epon. Semithin sections (1 µm) were stained with Methylene Blue for light-microscopic analysis. Thin sections (around 60 nm) were stained with uranyl acetate and lead citrate, then viewed by TEM (Philips CM100).

### RNA extraction and Quantitative PCR

Five month old wt and *Et(kita:GalTA4,UAS:mCherry)hzm1;Tg(UAS:eGFP-H-RASV12)io6* also in the *tp53-/-* background (also with tumor) were gamma irradiated or not with 12 Gy (Santoriello et al., 2009). After 16 hrs caudal fins were collected and omogenized in RLT buffer, and total RNA was extracted with RNeasy mini kit (Quiagen). After treatement with turbo DNAse (Ambion), RNA quantity was determined by spectrophotometer. cDNA was synthetized from total RNA (100 ng) using SuperScript VILO (Invitrogen) according to the manufacturer's instructions. Quantitative Real-time PCR was performed with LightCycler® 480 II thermocycler (Roche) using the SYBR Green I Master kit (Roche). cDNA (1 µl on 20 µl) was amplified in triplicate in a reaction volume of 25 µl. Primers, designed using Primer 3, were: p21 forward: 5′-ATGCAGCTCCAGACAGATGA-3′, p21 reverse: 5′-CGCAAACAGACCAACATCAC-3′, mdm2 forward: 5′-CCGAGGCAGACTACTGGAAG-3′, mdm2 reverse: 5′-GGAAGGTTGTGTTGGGAGTT-3′, β-actin forward: 5′- CGAGCAGGAGATGGGAACC-3′, β-actin reverse: 5′- CAACGGAAACGCTCATTGC-3′. The conditions for the reactions were the following: 10 min at 95°C, followed by 40 cycles of 15 s at 95°C and 60 s at 60°C. For any sample the expression level was analyzed using Q-Gene® software, which expresses data as mean normalized expression. All the genes were normalized to expression of β-actin as an endogenous control.

### Transplants and mosaic analysis

Genetic mosaics were generated by transplantation following standard protocols [45]. Donor *kita-GFP-RasV12* embryos were allowed to develop until shield stage. Approximately 20–30 cells were then transplanted into age-matched *nacre* host embryos. Embryos were raised to adulthood and checked for tumor formation.

For the re-expression of *mitfa* into *nacre*-GFP-RAS line we injected a T*2MUAMCS* plasmid containing the *mitfa cDNA* in combination with Tol2 transposase.

For somatic expression, the *UAS-eGFP-RasV12* plasmid was injected in combination with the transposase (25nl plasmid+35nl transposase mRNA) at one cell stage into the *kita*:Gal4 and *mitfa:*Gal4 embryos [46]. Larvae were sorted for eGFP-RasV12 expression. We counted the number of fish displaying melanocytic lesions at 4dpf, 15dpf and 1month.

## Supporting Information

Figure S1 **Range of caudal fin phenotypes in kita-GFP-RAS juvenile fish.** a, f, k) control fish (*kita*-GFP line). b–e, g–j, l–o) *kita*-GFP-RAS line. a–e) 15 dpf; f–k)18–22 dpf (pre-metamorphosis); j–o) 21–28 dpf (postmetamorphosis). Tumors are present in e, j, o. Calibration bar  = 1mm
(TIF)

Figure S2 **Histology of melanoma. a–c)** H&E staining of representative tail regions of kita-GFP-RAS fish at the ages indicated. d–e) Representative electromicrographs of a hyperpigmented (d) and a hypopigmented (e) melanoma. f) *in situ hybridization* for *dopachromo tautomerasis (dct)* in a cryostat section of a tail hypopigmented melanoma from a 1 month old transgenic zebrafish. Calibration bars  = 100 µm for a,b,c,f; 0.5 mm for d and e.
(TIF)

Figure S3 **Expression of kita-HRASV12 in mitfa-/- (nacre) background is unable to rescue melanocyte survival.** a) Migratory neural crest are the only cells expressing GFP-HRAS in 3 dpf *kita*-GFP-RAS embryos in a *nacre* background (b). Compare with fully differentiated, transformed tail melanocytes (c) in a 3 dpf *kita*-GFP-RAS embryo in a AB background (d). e) 21 dpf *nacre* x *kita*-GFP-RAS fish is devoid of melanocytes, as opposed to AB x *kita*-GFP-RAS fish (f), which has increased pigmentation. Calibration bar 100 mm for a–d; 2 mm for e–f.
(TIF)

Figure S4 **Similarities and differences between mitfa-GFP-RAS and kita-GFP-RAS transgenic lines.** a–b, e–f) Similar expression pattern, but different number of GFP-RAS migrating melanocyte progenitors at 32 hpf in mitfa-GFP (a,b) and kita-GFP (e,f) double transgenic zebrafish. c) Normal pigmentation in a 5 dpf mitfa-GFP-RAS larva. d) The only melanoma that developed in mitfa-GFP-RAS fish by three months of age (n = 25). g–h) Other cell types expressing GFP under the kita promoter. Melanocytes and mucous cells (arrows) in kita-GFP 3dpf larvae (g). Flat epithelial cells (enlarged in inset), present in the fins of kita-GFP 3 dpf larvae (k). These cells do not change in number or size upon expression of HRAS. i). Western Blot analysis of protein extracts from double transgenic fish at the age indicated in the upper lane. Increased levels of Ras in kita:GFP-RAS versus mita:GFP-RAS fish. Calibration bars: 50 µm in a–b;e–n; 2 mm in c–d.
(TIF)
