# Supplementary figures and images for: Kita Driven Expression of Oncogenic HRAS Leads to Early Onset and Highly Penetrant Melanoma in Zebrafish
Source: PLoS One. 2010 Dec 10;5(12):e15170. doi: 10.1371/journal.pone.0015170 (PMC3000817; doi:10.1371/journal.pone.0015170)

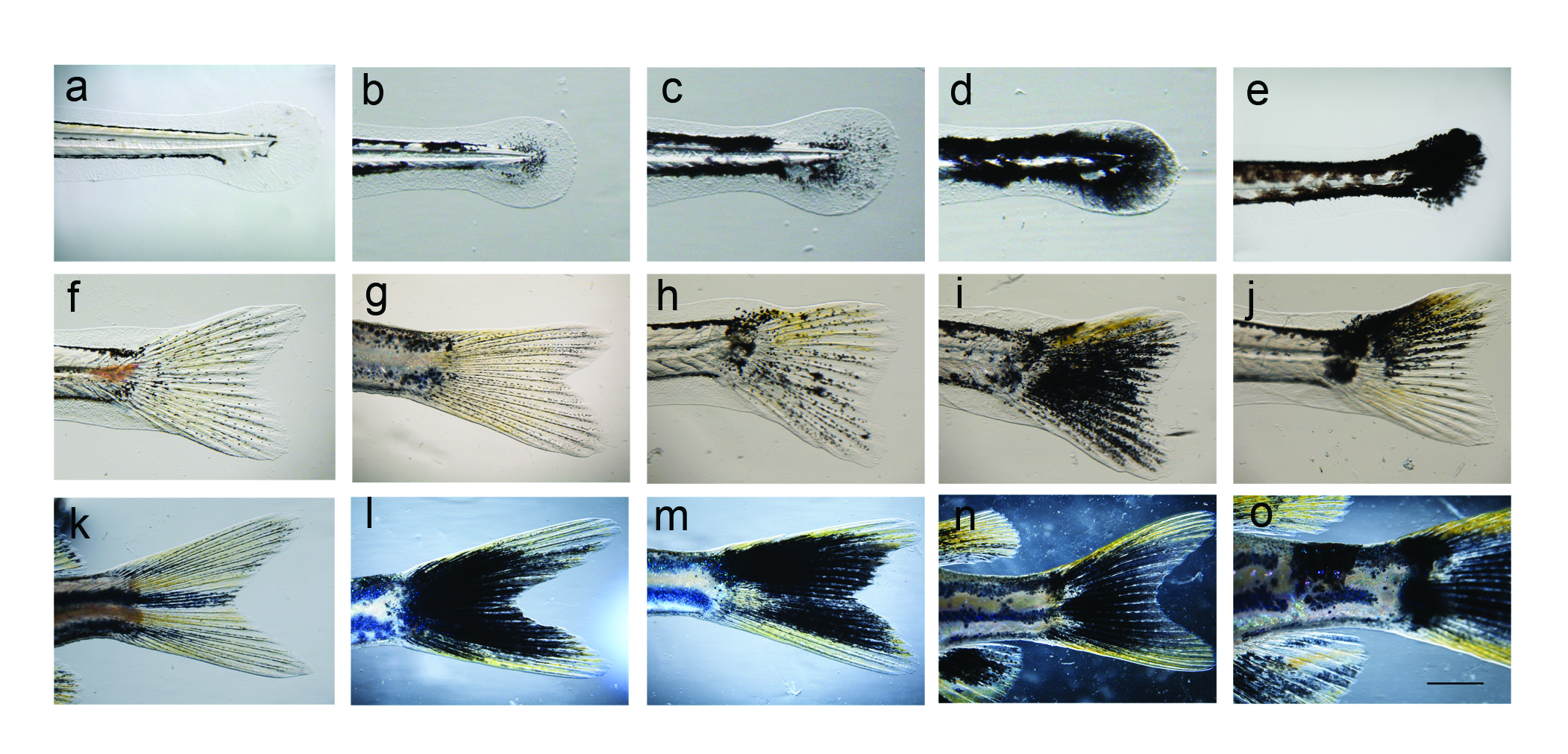

Supplement: Figure S1 — Range of caudal fin phenotypes in kita-GFP-RAS juvenile fish. a, f, k) control fish (kita-GFP line). b–e, g–j, l–o) kita-GFP-RAS line. a–e) 15 dpf; f–k)18–22 dpf (pre-metamorphosis); j–o) 21–28 dpf (postmetamorphosis). Tumors are present in e, j, o. Calibration bar = 1mm (TIF) [file pone.0015170.s001.tif]

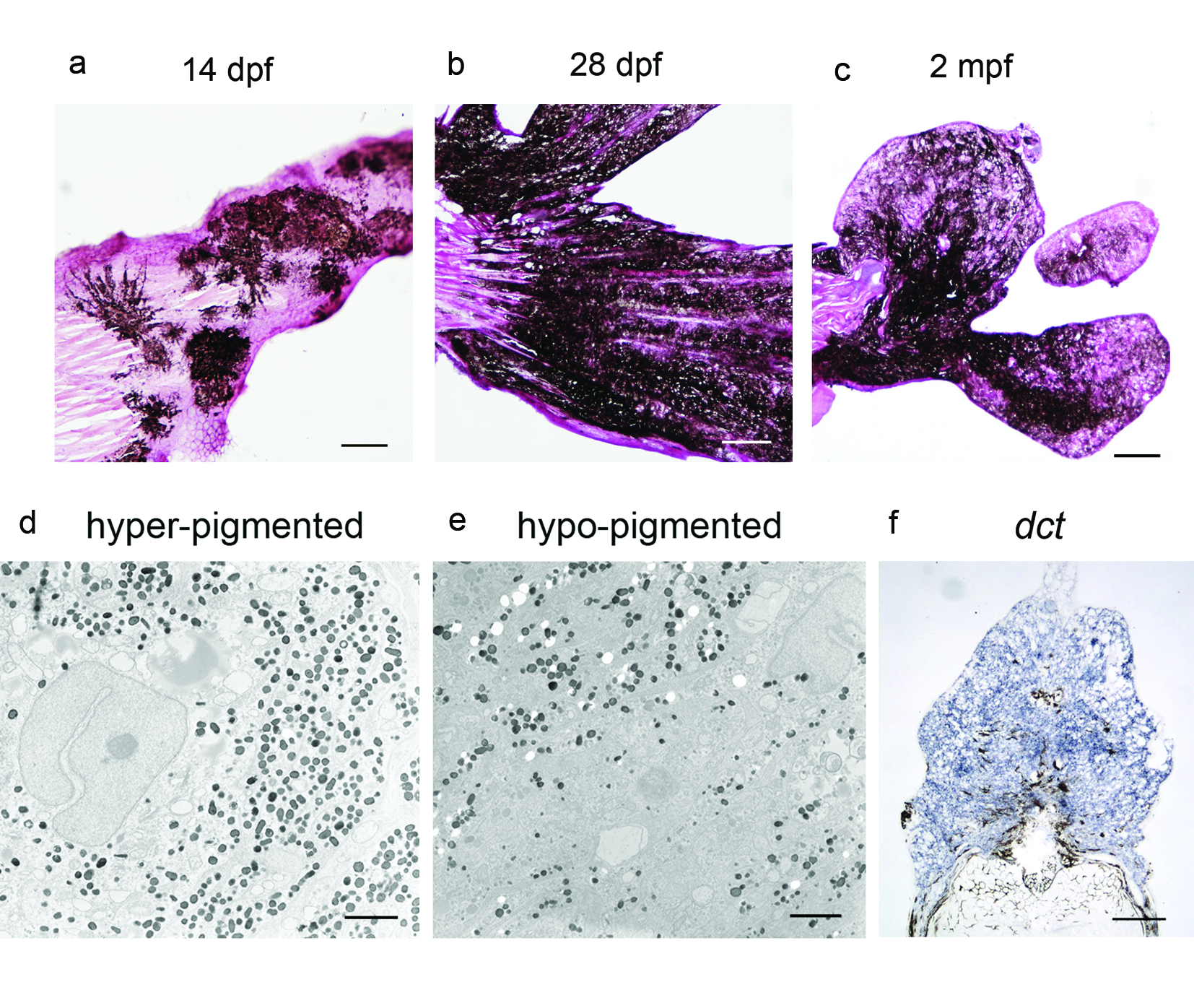

Supplement: Figure S2 — Histology of melanoma. a–c) H&E staining of representative tail regions of kita-GFP-RAS fish at the ages indicated. d–e) Representative electromicrographs of a hyperpigmented (d) and a hypopigmented (e) melanoma. f) in situ hybridization for dopachromo tautomerasis (dct) in a cryostat section of a tail hypopigmented melanoma from a 1 month old transgenic zebrafish. Calibration bars = 100 µm for a,b,c,f; 0.5 mm for d and e. (TIF) [file pone.0015170.s002.tif]

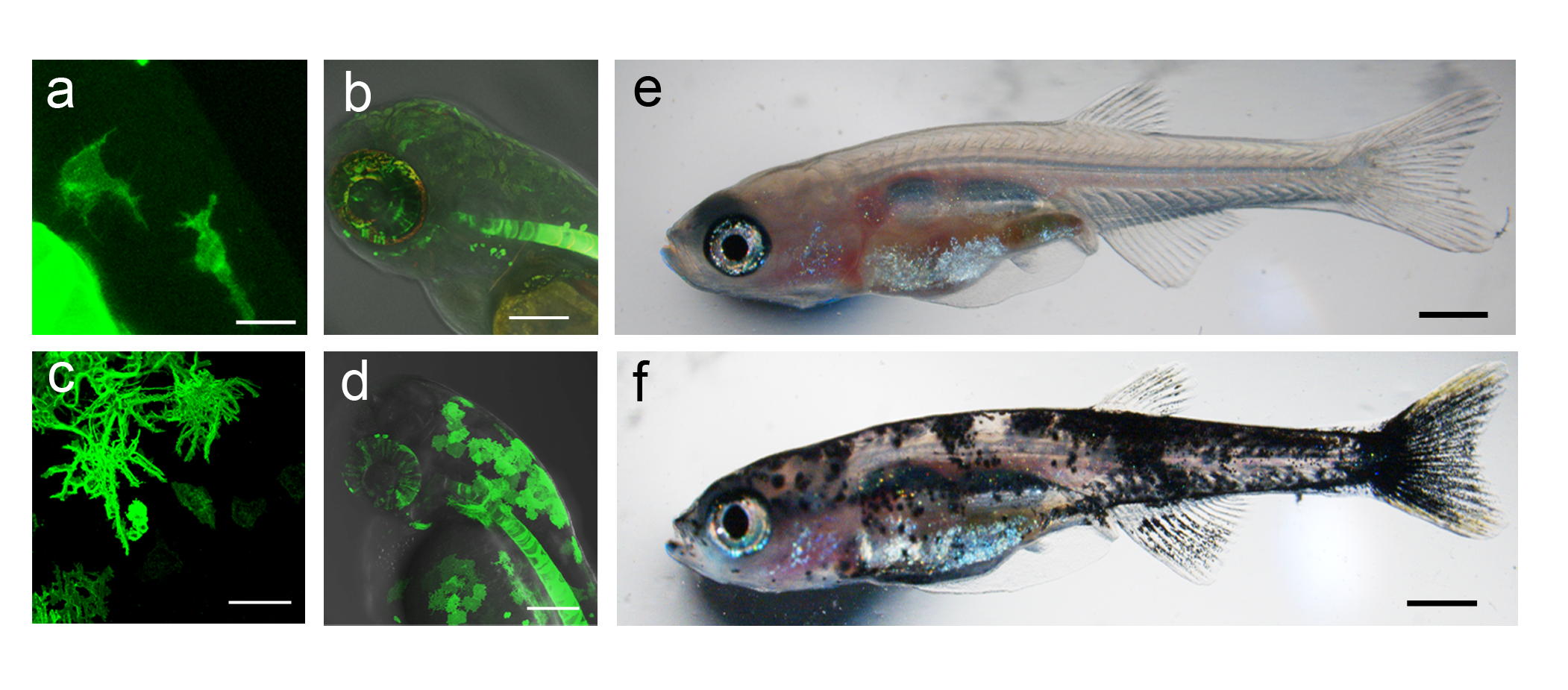

Supplement: Figure S3 — Expression of kita-HRASV12 in mitfa-/- (nacre) background is unable to rescue melanocyte survival. a) Migratory neural crest are the only cells expressing GFP-HRAS in 3 dpf kita-GFP-RAS embryos in a nacre background (b). Compare with fully differentiated, transformed tail melanocytes (c) in a 3 dpf kita-GFP-RAS embryo in a AB background (d). e) 21 dpf nacre x kita-GFP-RAS fish is devoid of melanocytes, as opposed to AB x kita-GFP-RAS fish (f), which has increased pigmentation. Calibration bar 100 mm for a–d; 2 mm for e–f. (TIF) [file pone.0015170.s003.tif]

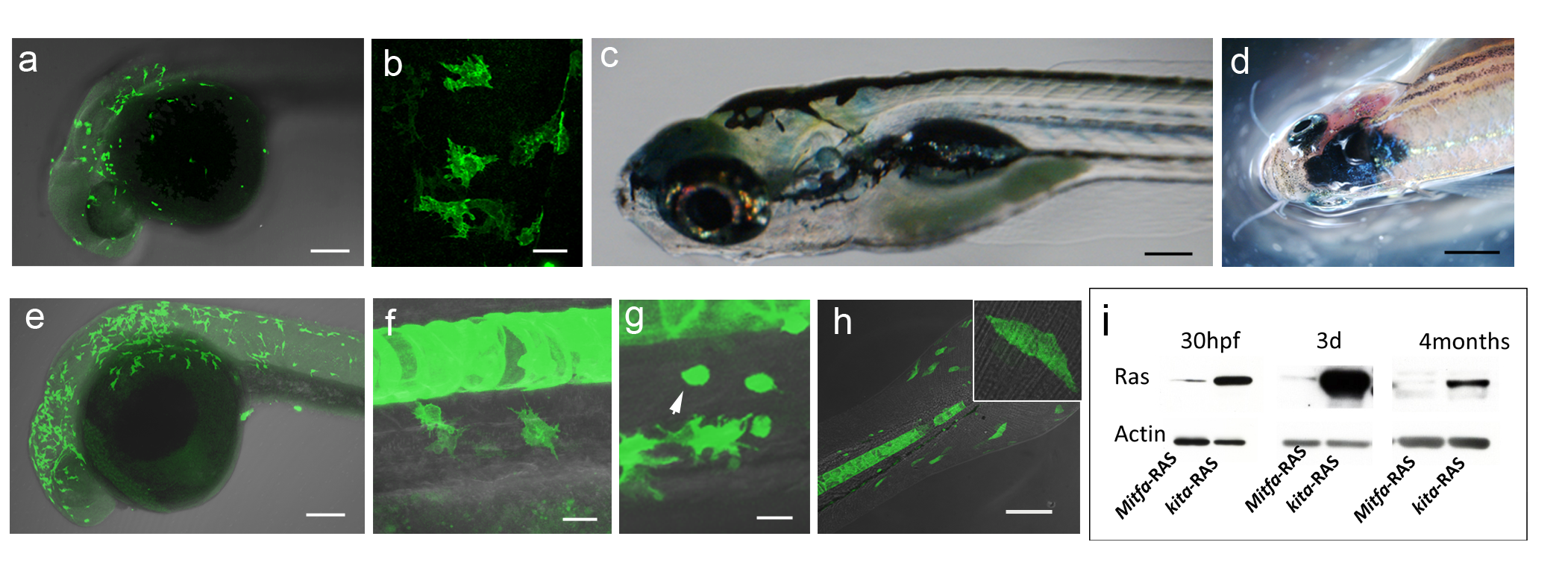

Supplement: Figure S4 — Similarities and differences between mitfa-GFP-RAS and kita-GFP-RAS transgenic lines. a–b, e–f) Similar expression pattern, but different number of GFP-RAS migrating melanocyte progenitors at 32 hpf in mitfa-GFP (a,b) and kita-GFP (e,f) double transgenic zebrafish. c) Normal pigmentation in a 5 dpf mitfa-GFP-RAS larva. d) The only melanoma that developed in mitfa-GFP-RAS fish by three months of age (n = 25). g–h) Other cell types expressing GFP under the kita promoter. Melanocytes and mucous cells (arrows) in kita-GFP 3dpf larvae (g). Flat epithelial cells (enlarged in inset), present in the fins of kita-GFP 3 dpf larvae (k). These cells do not change in number or size upon expression of HRAS. i). Western Blot analysis of protein extracts from double transgenic fish at the age indicated in the upper lane. Increased levels of Ras in kita:GFP-RAS versus mita:GFP-RAS fish. Calibration bars: 50 µm in a–b;e–n; 2 mm in c–d. (TIF) [file pone.0015170.s004.tif]
